# MRI evaluation of vesical imaging reporting and data system for bladder cancer after neoadjuvant chemotherapy

**DOI:** 10.1186/s40644-024-00696-6

**Published:** 2024-04-08

**Authors:** Xinxin Zhang, Yichen Wang, Yilin Wang, Jie Zhang, Jin Zhang, Lianyu Zhang, Sicong Wang, Jianzhong Shou, Yan Chen, Xinming Zhao

**Affiliations:** 1 Department of Diagnostic Radiology, National Cancer Center/National Clinical Research Center for Cancer/Cancer Hospital, Chinese Academy of Medical Sciences and Peking Union Medical College, No. 17 Panjiayuan Nanli, Chaoyang District, Beijing, 100021 China; 2 GE Healthcare, MR Research China, Beijing, 100176 China; 3 Department of Urology, National Cancer Center/National Clinical Research Center for Cancer/Cancer Hospital, Chinese Academy of Medical Sciences and Peking Union Medical College, No.17 Panjiayuan Nanli, Chaoyang District, Beijing, 100021 China

**Keywords:** Urinary bladder neoplasms, MRI, Neoadjuvant chemotherapy, Neoplasm staging

## Abstract

**Background:**

The Vesical Imaging-Reporting and Data System (VI-RADS) has demonstrated effectiveness in predicting muscle invasion in bladder cancer before treatment. The urgent need currently is to evaluate the muscle invasion status after neoadjuvant chemotherapy (NAC) for bladder cancer. This study aims to ascertain the accuracy of VI-RADS in detecting muscle invasion post-NAC treatment and assess its diagnostic performance across readers with varying experience levels.

**Methods:**

In this retrospective study, patients with muscle-invasive bladder cancer who underwent magnetic resonance imaging (MRI) after NAC from September 2015 to September 2018 were included. VI-RADS scores were independently assessed by five radiologists, consisting of three experienced in bladder MRI and two inexperienced radiologists. Comparison of VI-RADS scores was made with postoperative histopathological diagnosis. Receiver operating characteristic curve analysis (ROC) was used for evaluating diagnostic performance, calculating sensitivity, specificity, and area under ROC (AUC)). Interobserver agreement was assessed using the weighted kappa statistic.

**Results:**

The final analysis included 46 patients (mean age: 61 years ± 9 [standard deviation]; age range: 39–70 years; 42 men). The pooled AUC for predicting muscle invasion was 0.945 (95% confidence interval (CI): 0.893–0.977) for experienced readers, and 0.910 (95% CI: 0.831–0.959) for inexperienced readers, and 0.932 (95% CI: 0.892–0.961) for all readers. At an optimal cut-off value ≥ 4, pooled sensitivity and specificity were 74.1% (range: 66.0–80.9%) and 94.1% (range: 88.6–97.7%) for experienced readers, and 63.9% (range: 59.6–68.1%) and 86.4% (range: 84.1–88.6%) for inexperienced readers. Interobserver agreement ranged from substantial to excellent between all readers (k = 0.79–0.92).

**Conclusions:**

VI-RADS accurately assesses muscle invasion in bladder cancer patients after NAC and exhibits good diagnostic performance across readers with different experience levels.

## Introduction

Bladder cancer ranks among the most frequently diagnosed cancers globally [1], with approximately 25% of cases being muscle-invasive bladder cancer (MIBC) [2]. The established standard of care for MIBC involves neoadjuvant chemotherapy (NAC) followed by radical cystectomy [3]. However, the incidence of adverse events linked to radical cystectomy is notably high, ranging from 49.1% to 69.0% [4–6]. Additionally, certain elderly patients, due to age-related functional decline or comorbidities, may not be suitable candidates for radical cystectomy. Consequently, there is a growing focus on bladder preservation therapies for MIBC [7–9]. Studies have shown that bladder-sparing therapies can yield favorable oncologic outcomes, with 5-year survival rates ranging from 64 to 87% for patients responding well to NAC [10–12]. Thus, it is imperative to determine the presence of a residual tumor and its invasion into the muscularis propria for local restaging following neoadjuvant therapy.

For restaging bladder cancer post-NAC, transurethral resection of bladder tumor (TURBT) is a commonly employed method [13, 14]. However, TURBT's reliability in post-NAC clinical restaging is compromised, as it falsely downstages 32% of patients when compared to the final pathology results from radical cystectomy [15, 16]. Consequently, there is an urgent need to establish a precise approach for post-NAC clinical restaging in MIBC patients.

Magnetic resonance imaging (MRI) is widely utilized for locoregional staging of bladder cancer due to its non-invasive nature, reproducibility, and excellent soft tissue contrast [17, 18]. In 2018, the Vesical Imaging Reporting and Data System (VI-RADS) was introduced to standardize imaging and reporting [19]. Numerous studies have confirmed VI-RADS's robust diagnostic performance in assessing the risk of muscular propria invasion in primary bladder cancer [20–24]. While the neoadjuvant chemotherapy VI-RADS scoring system has been developed to evaluate treatment response in MIBC patients, most research has focused on untreated bladder cancer cases. Hence, there is a growing interest in determining VI-RADS's reliability for restaging after NAC [25, 26]. To our knowledge, only one study has demonstrated VI-RADS's favorable diagnostic performance in post-treatment patients undergoing TURBT, partial cystectomy, NAC, and intravesical instillation therapy. In this study, VI-RADS exhibited a sensitivity of 91.7% (95% CI: 61.5, 99.8) and a specificity of 89.5% (95% CI: 66.9, 98.7) [27]. However, it's crucial to note that only four patients received NAC alone in this study. Furthermore, the diagnostic performance and inter-reader reliability of VI-RADS in evaluating muscle invasion in patients undergoing NAC treatment remain unknown.

Therefore, considering the urgent requirement for local tumor reassessment post-NAC, this study aimed to investigate VI-RADS's performance in evaluating muscle invasion in bladder cancer patients after NAC. Additionally, inter-reader reproducibility was evaluated among readers with varying levels of experience.

## Methods and materials

The Ethics Committee of our hospital granted approval for this retrospective study, and a waiver for written informed consent was obtained.

### Patients

Patients with bladder cancer participating in the prospective, single-center, phase II trial (NCT02861196) from September 2015 to September 2018 were retrospectively analyzed. Trial participants were clinically diagnosed with MIBC (clinical T2-4aN0M0) confirmed by cystoscopic biopsy (not TURBT) and imaging. Prior to NAC, all patients underwent multiparametric MRI. Two radiologists (Y.C.W. and J.Z., with 9 and 12 years of radiology experience, respectively) utilized the VI-RADS scoring system to assess three image sequences: T2-weighted imaging (T2WI), diffusion-weighted imaging (DWI), and dynamic contrast-enhanced (DCE) imaging. VI-RADS scores were assigned following the criteria outlined in the literature [19]. In cases of disagreement between the two readers, consensus was reached through discussion. A meta-analysis has indicated that using VI-RADS ≥ 4 as a cutoff value yields high accuracy in evaluating muscular infiltration [28]. Consequently, tumors with a VI-RADS score of less than 4 on pre-NAC MRI were excluded from the present study.

The NAC regimen comprised gemcitabine and cisplatin. Subsequent to NAC, patients underwent one of the following treatment options: TURBT plus concurrent chemoradiotherapy or bacillus Calmette-Guerin instillation, partial cystectomy with pelvic lymphadenectomy, or radical cystectomy plus pelvic lymphadenectomy. For tumors subjected to TURBT, a section of detrusor muscle tissue at the tumor base underwent individual examination for histopathology to ascertain detrusor muscle invasion.

**Fig. 1:**
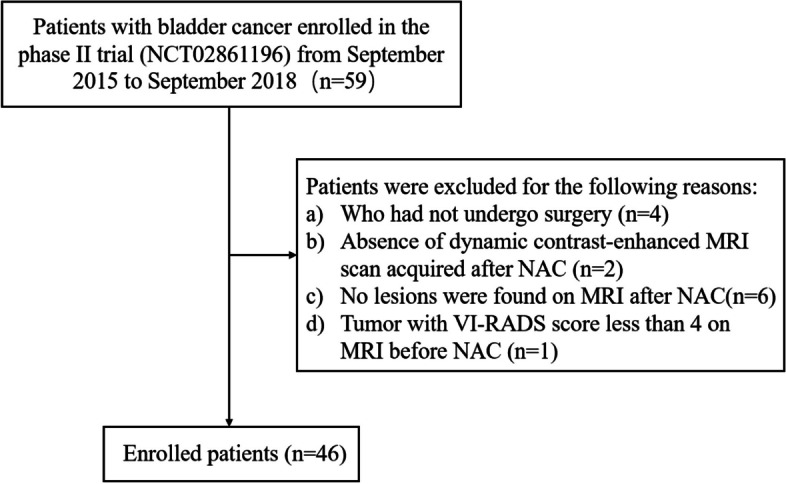
Flowchart shows the patient selection process in the study

Inclusion criteria were: (a) urothelial carcinoma without carcinoma in situ, confirmed by tumor plus random biopsies; (b) no invasion of the ureteral orifice; (c) all patients underwent pre- and post-NAC MRI examination; (d) no history of other malignant tumors. Exclusion criteria were: (a) patients not undergoing surgery; (b) absence of contrast-enhanced MRI scan after NAC; (c) no lesions detected on post-NAC MRI examination; (d) tumor with VI-RADS score less than 4 on pre-NAC MRI. Figure 1 illustrates the study flowchart.

## MRI examination

The MR examination was conducted using a 3.0 T scanner (Discovery 750, GE Healthcare, Milwaukee) with an eight-channel phased array body coil in the supine position. The MRI sequences included two-dimensional fast spin-echo or PROPELLER T2-weighted sequences in axial, coronal, and sagittal planes without fat suppression, axial fat-suppressed T2-weighted imaging, axial DWI with b values of 0 and 1000 s/mm^2^, and axial fat-suppressed DCE imaging. DCE imaging was performed following intravenous injection of 0.1 mL per kilogram of body weight of gadopentetate dimeglumine (Magnevist; Bayer Schering Pharma) at a rate of 2 ml/s. The early phase of DCE imaging was acquired 20 s after contrast material injection, followed by four consecutive sequences taken every 30 s. Detailed MRI parameters are provided in Table 1.

**Table 1:** Multiparametric MRI protocol at 3.0 T

Parameters	T2WI	DWI	DCE
Repetition time (ms)	5043	2288	3964
Echo time (ms)	102	58.4	1848
No. of echo trains per section	21	1	1
Matrix size	320 × 256	128 × 160	288 × 224
Field of view (cm × cm)	20 × 20	38 × 38	
Slice thickness (mm)	3	5	3
Interslice gap (mm)	0.3	0.3	0.3
Number of excitations	2	4	1
Acquisition time (sec)	146	32	74
b-value (sec/mm^2^)		0, 1000	

To achieve moderate bladder filling, patients were instructed to empty their bladders 1 h before the scan and then consume 500 ml (mL) of water. Patients without contraindications to antispasmodics received 1 mL scopolamine butylbromide intramuscularly 10 min before MRI examinations.

### Imaging analysis

Three sequences (T2WI, DWI, and DCE-MRI) were independently evaluated by five radiologists at 2-week intervals between each sequence. Three chief physicians (L.Y.Z., J.Z., and Y.C., with 21, 20, and 28 years of experience, respectively, in pelvic imaging) were classified as "experienced" readers, while two residents (Y.L.W. and X.X.Z., with 2 and 3 years of experience, respectively, in pelvic imaging) were categorized as "inexperienced" readers. Each radiologist underwent training by reviewing more than 50 multiparametric MRI cases of bladder cancer before formal review. In cases with multiple lesions, a radiologist (X.M.Z., with 36 years of experience in pelvic imaging) not involved in the VI-RADS evaluation marked the lesion. The radiologist selected the lesions with the highest T stage by reviewing surgical records, pathological results, and imaging. For tumors with the same T stage, the largest one was chosen. Ultimately, a VI-RADS score reflecting the overall risk of detrusor muscle invasion was assigned to each patient. Scoring was conducted using the five-point scoring system in strict accordance with VI-RADS criteria described in the literature [19]. All readers were blinded to the patient's clinical history, type of surgery, and pathological results.

### Statistical analysis

Categorical data were presented as numbers and proportions, while continuous variables were shown as mean ± standard deviation. Inter-reader agreement was assessed using weighted kappa statistics, where a kappa value up to 0.20 indicated slight agreement, 0.21–0.40 denoted fair agreement, 0.41–0.60 suggested moderate agreement, 0.61–0.80 indicated substantial agreement, and 0.81 or greater represented excellent agreement. Receiver operating characteristic curve (ROC) analysis was employed to assess the performance of VI-RADS scores in predicting muscle-invasive tumors. The optimal cutoff scores of VI-RADS were determined based on the Youden index. Parameters such as the area under ROC (AUC), sensitivity, specificity, accuracy, positive predictive value, and negative predictive value were estimated. Pooled (reader-averaged) diagnostic outcomes and AUC of VI-RADS were determined using multi-reader multi-case ROC curve analysis [29]. A Delong test was conducted to compare ROC curves. Statistical analyses were carried out using SPSS software (version 20; IBM, Armonk, NY), MedCalc software 15.8 (MedCalc Software bvba, Ostend, Belgium), and R version 3.4.3. A two-tailed p-value less than 0.05 was considered statistically significant.

## Results

### Patient and lesion characteristics

A total of 59 patients with MIBC (clinical T2-4aN0M0) were initially considered for inclusion. Prior to NAC, all patients were evaluated by MRI, computed tomography urography of the thoracic and abdominopelvic cavities and underwent multidisciplinary team discussions with clinical T staging defined. Exclusions were made for the following: (a) 4 patients who did not undergo surgery; (b) 2 patients without a post-NAC contrast-enhanced MRI scan; (c) 6 patients with no lesions detected on post-NAC MRI; and (d) 1 patient with a tumor VI-RADS score less than 4 on pre-NAC MRI. Consequently, the final analysis included 46 patients (mean age: 61 years ± 9 [standard deviation]; age range: 39–70 years; 42 men). Figure 1 illustrates the patient selection process and outcomes. Among the 46 NAC-treated patients, 24 (52%) underwent TURBT plus concurrent chemoradiotherapy or Bacillus Calmette-Guerin, 4 (9%) underwent partial cystectomy plus pelvic lymphadenectomy, and 18 (39%) underwent radical cystectomy.

Pathological findings indicated that 21 out of 46 patients (46%) had MIBC (T2 or higher), while 25 out of 46 (54%) had NMIBC (T1 or lower). Of these patients, 32 (70%) had a single tumor, and 14 (30%) had multiple tumors. Submucosal linear enhancement on DCE was observed in 11 out of 46 (24%) tumors. Among these 11 tumors, continuous submucosal linear enhancement was noted in 6 tumors (55%) with pathologic confirmation of non-muscle-invasive bladder cancer. Of the remaining 5 tumors with discontinuous submucosal linear enhancement, one tumor (20%) was non-muscle-invasive, and four tumors were muscle-invasive. The median time interval between post-NAC MRI and pathologic staging assessment through surgery was 14 days (range: 8 to 37 days). Further details on patient and lesion characteristics are summarized in Table 2.

**Table 2:** Clinical and Pathologic Characteristics of Patients (*n* = 46)

Characteristics	MIBC (*n* = 21)	NMIBC (*n* = 25)
Age, year		
Mean ± Standard Deviation	63 ± 9	60 ± 10
Range	45–77	29–72
Gender		
Male	20 (95)	22 (88)
Female	1 (5)	3 (12)
Number of lesions		
Solitary	19 (90.0)	13 (52)
Multiple	2 (10.0)	12 (48)
Submucosal Linear Enhancement		
Yes	4 (19)	7 (28)
No	17 (81)	18 (72)
Primary or recurrent tumors		
Primary	16 (76)	21 (84)
Recurrent	5 (24)	4 (16)
Histologic grade		
Low grade	3 (14)	6 (24)
High grade	18 (86)	19 (76)
NAC cycles		
2	5 (23.8)	7 (28)
3	10 (47.6)	11 (44)
4	6 (28.6)	7 (28)
Treatment method		
NAC + Radical cystectomy	13 (62)	5 (20)
NAC + Partial cystectomy	3 (14)	1 (4)
NAC + TURBT	5(24)	19 (76)

Numbers in parentheses are percentages

*TURBT* Transurethral resection of bladder tumor, *MIBC* Muscle-invasive bladder cancer, *NAC* Neoadjuvant chemotherapy, *NMIBC* Non–muscle-invasive bladder cancer

### Performance of overall VI-RADS scores for diagnosing muscle invasion

**Fig. 2:**
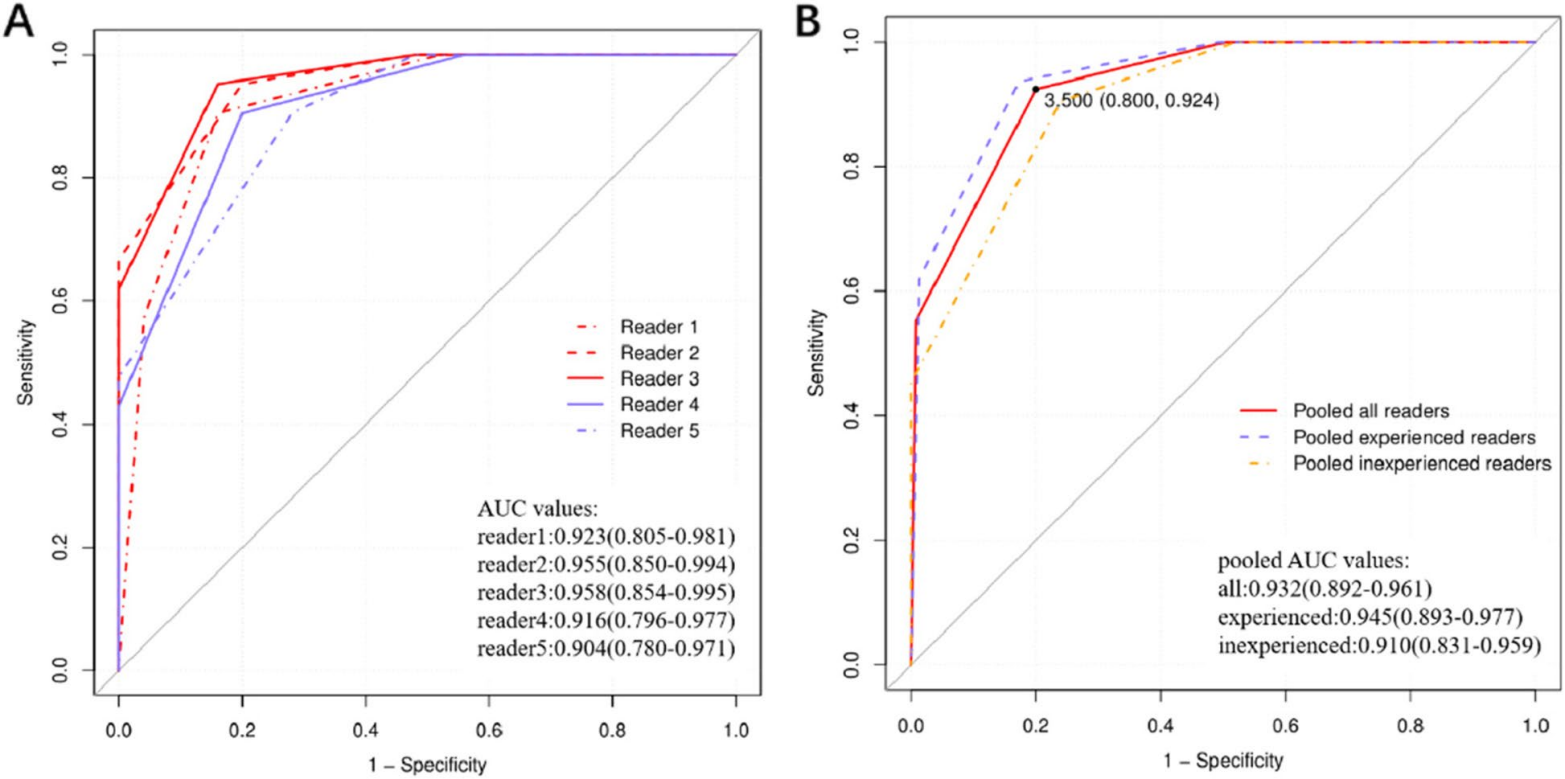
Receiver operating characteristic curve of Vesical Imaging Reporting and Data System score for predicting muscle invasion of bladder cancer by the five readers (**A**) and pooled readers (**B**) with different levels of experience. AUC = area under the receiver operating characteristic curve

**Fig. 3:**
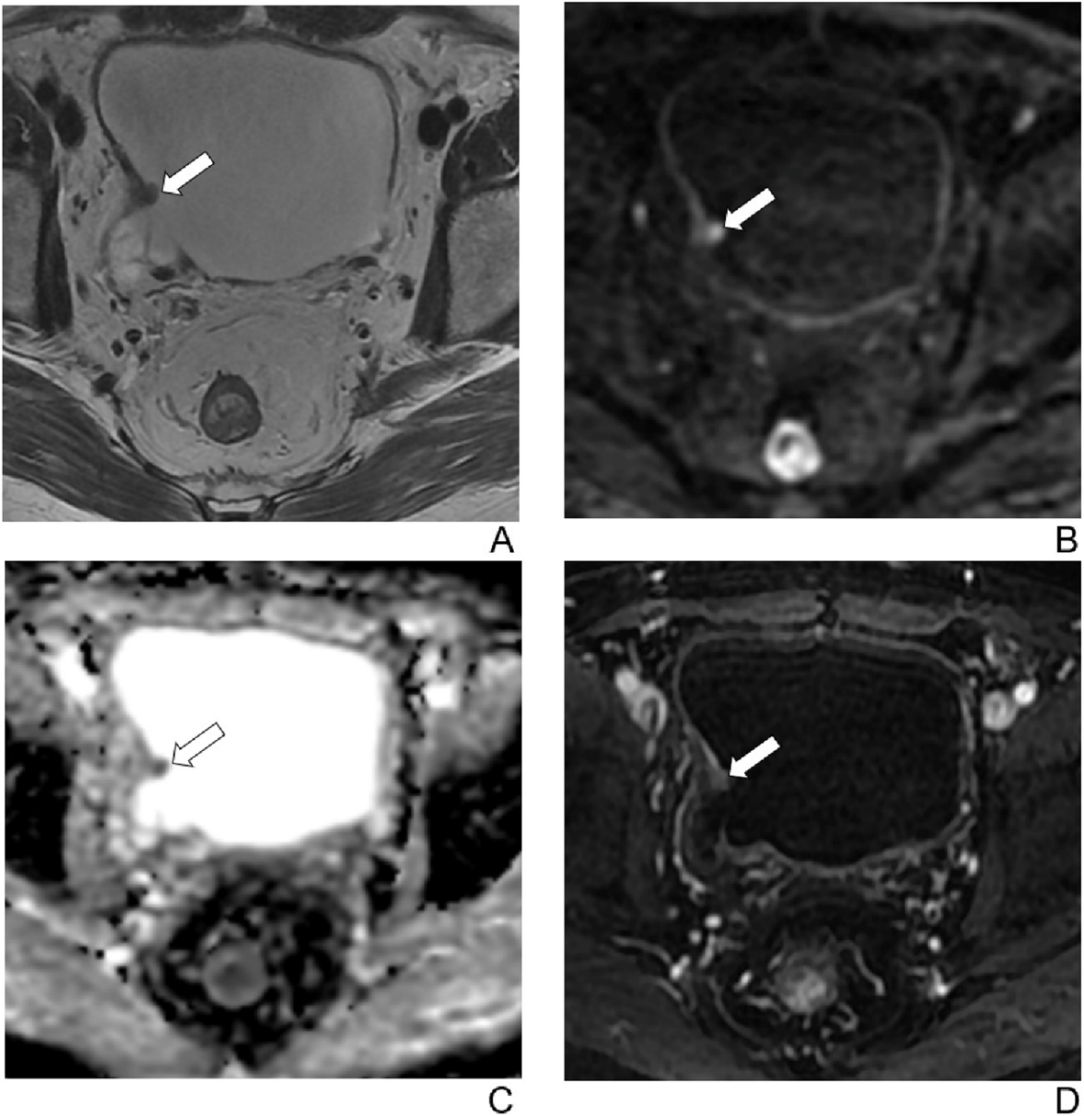
A 69-year-old man with hematuria for 2 months. A biopsy revealed high-grade urothelial carcinoma. The patient underwent 2 cycles of neoadjuvant chemotherapy followed by transurethral resection of the bladder tumor. **A** Axial T2-weighted image shows an exophytic tumor on the right side of bladder, < 1 cm in greatest dimension. Disruption of low-signal-intensity muscularis propria is unclear. **B** Axial diffusion-weighted image (b = 1000) and (**C**) apparent diffusion coefficient images show uninterrupted intermediate signal-intensity muscularis propria. **D** Axial contrast-enhanced image shows uninterrupted low-signal-intensity muscularis propria. The final VI-RADS score was 1 for all readers. Histopathology confirmed a non-muscle-invasive bladder cancer

**Fig. 4:**
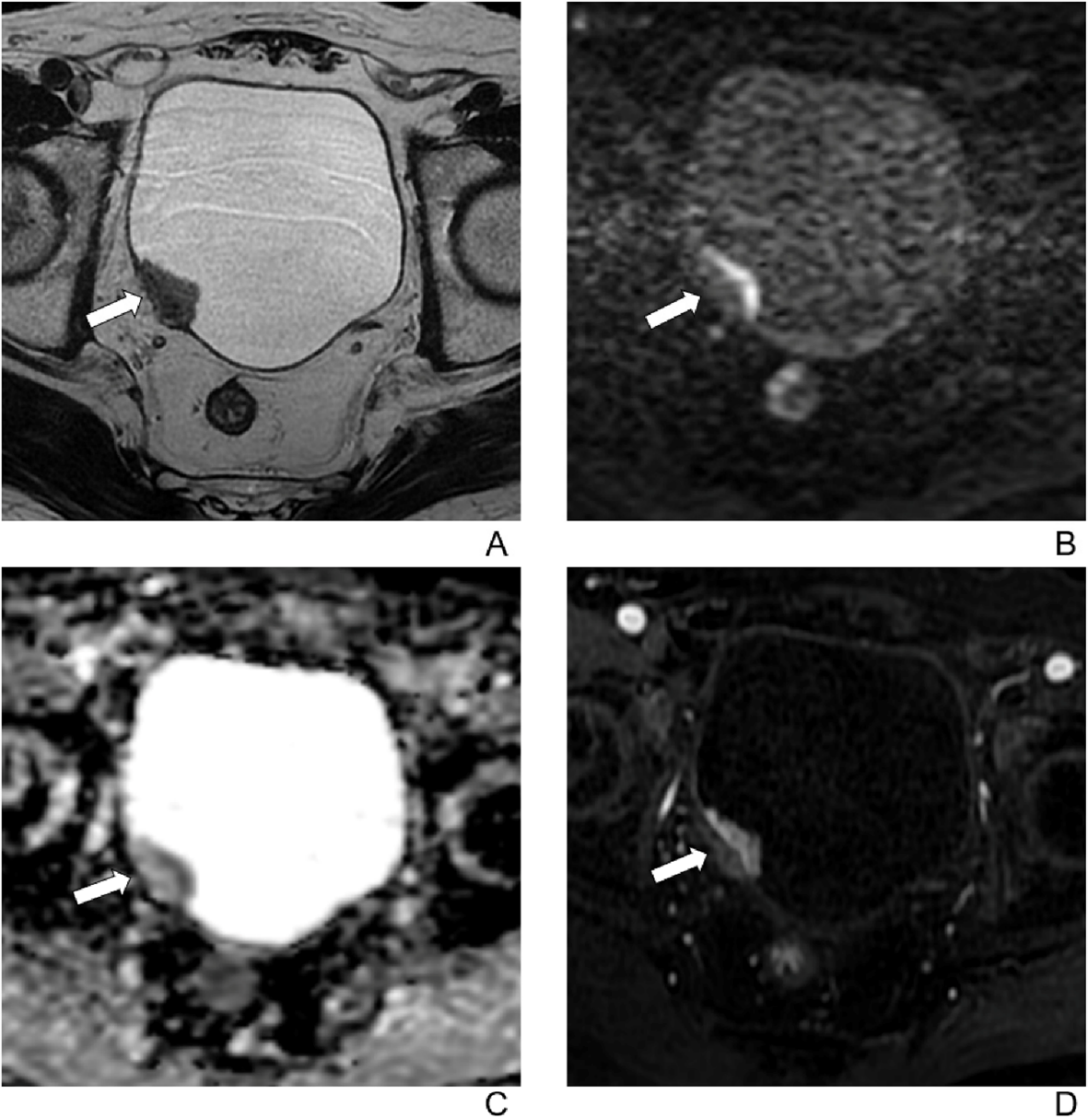
A 63-year-old man with hematuria for 6 months. A biopsy revealed high-grade urothelial carcinoma. The patient underwent 2 cycles of neoadjuvant chemotherapy followed by transurethral resection of the bladder tumor. **A** Axial T2-weighted image shows an exophytic tumor extending to extravesical fat on the right side of bladder. **B** Axial diffusion-weighted image (b = 1000) shows high-signal-intensity sessile tumor with low-signal-intensity thickened inner layer. **C** Apparent diffusion coefficient image shows low-signal-intensity sessile tumor with high-signal-intensity thickened inner layer. **D** Axial contrast-enhanced image shows early enhancement tumor with continuous submucosal linear enhancement (arrows). The final VI-RADS score was 2 for all readers. Histopathology confirmed a non-muscle-invasive bladder cancer

**Fig. 5:**
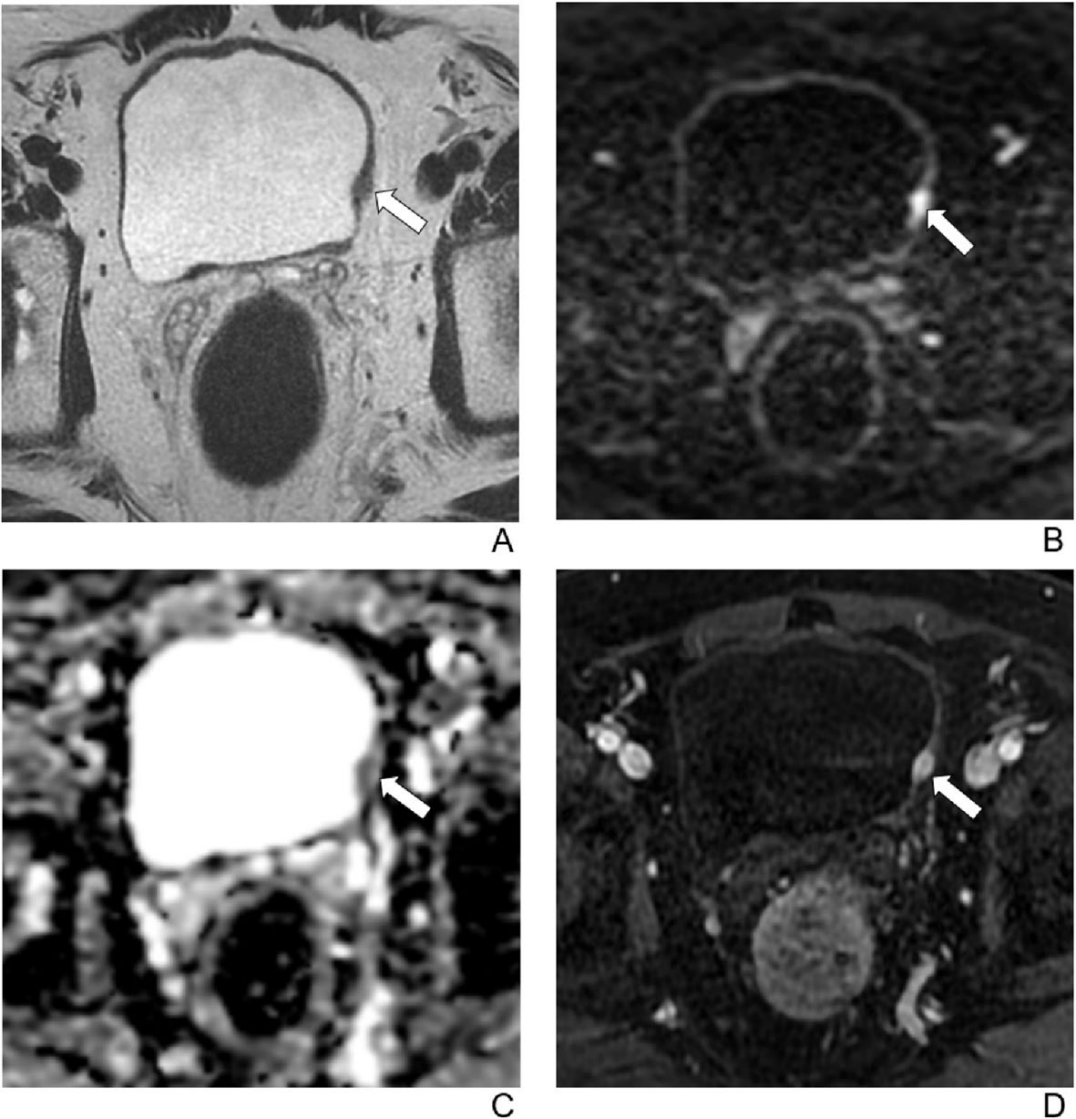
A 73-year-old man with hematuria for 6 months. A biopsy revealed high-grade urothelial carcinoma. The patient underwent 3 cycles of neoadjuvant chemotherapy followed by transurethral resection of the bladder tumor. **A** Axial T2-weighted image shows an endophytic tumor extending to extravesical fat on the left side of bladder. **B** Axial diffusion-weighted image (b = 1000) and (**C**) apparent diffusion coefficient images show interrupted intermediate signal-intensity muscularis propria. **D** Axial contrast-enhanced image shows tumor early enhancement extends to the entire bladder wall and to extravesical fat on the left side of bladder. The final VI-RADS score was 5 for all readers. Histopathology confirmed a muscle-invasive bladder cancer

The frequencies of MIBC for each VI-RADS score, as determined by each reader, are presented in Table 3. Across all readers, no cases scored as VI-RADS 1 or VI-RADS 2 exhibited MIBC. For experienced readers, MIBC was present in 11–18% of cases scored as VI-RADS 3, 55–70% of cases scored as VI-RADS 4, and 92–100% of cases scored as VI-RADS 5. Inexperienced readers identified MIBC in 18–29% of cases scored as VI-RADS 3, 56–67% of cases scored as VI-RADS 4, and 100.0% of cases scored as VI-RADS 5. The diagnostic performance of VI-RADS for each reader is detailed in Table 4 and Fig. 2. Across all readers, the AUC ranged from 0.904 to 0.955, with no statistically significant differences observed. The pooled AUC for predicting muscle invasion was 0.945 (95% CI: 0.893–0.977) for experienced readers, 0.910 (95% CI: 0.831–0.959) for inexperienced readers, and 0.932 (95% CI: 0.892–0.961) for all readers (*p* > 0.05). No statistical difference in the pooled AUC was found between experienced and inexperienced readers (*p* > 0.05). According to the Youden index, the optimal cut-off score for predicting muscle invasion was ≥ VI-RADS 4. The pooled sensitivity, specificity, accuracy, positive predictive value, and negative predictive value were as follows: for experienced readers, 93.7% (range, 90.5–95.2%), 82.7% (range, 72.0–88.6%), 87.7%, 82.0%, and 93.9%; for inexperienced readers, 90.5% (range, 77.4–97.3%), 76.0% (range, 61.8–86.9%), 82.6%, 76.0%, and 90.5%; and for all readers, 92.4% (range, 85.5–96.7%), 80.0% (range, 71.9–86.6%), 85.7%, 79.5%, and 92.6%. VI-RADS scores from representative examples are displayed in Figs. 3, 4 and 5.

**Table 3:** Frequency of MIBC and NMIBC for VI-RADS Score for Each Reader

Variable	VI-RADS 1	VI-RADS 2	VI-RADS 3	VI-RADS 4	VI-RADS 5
Reader1(Experienced)					
No.of cases	6	6	11	10	13
No.of cases with MIBC	0(0)	0(0)	2(18)	7(70)	12(92)
No.of cases with NMIBC	6(100)	6(100)	9(82)	3(30)	1(8)
Reader2(Experienced)					
No.of cases	6	7	8	11	14
No.of cases with MIBC	0(0)	0(0)	1(13)	6(55)	14(100)
No.of cases with NMIBC	6(100)	7(100)	7(88)	5(45)	0(0)
Reader3(Experienced)					
No.of cases	6	7	9	11	13
No.of cases with MIBC	0(0)	0(0)	1(11)	7(64)	13(100)
No.of cases with NMIBC	6(100)	7(100)	8(89)	4(36)	0(0)
Reader4(Inexperienced)					
No.of cases	6	5	11	15	9
No.of cases with MIBC	0(0)	0(0)	2(18)	10(67)	9(100)
No.of cases with NMIBC	6(100)	5(100)	9(82)	5(33)	0(0)
Reader5(Inexperienced)					
No.of cases	6	7	7	16	10
No.of cases with MIBC	0(0)	0(0)	2(29)	9(56)	10(100)
No.of cases with NMIBC	6(100)	7(100)	5(71)	7(44)	0(0)

Note—Values in parentheses are percentages of cases. *MIBC* Muscle-invasive bladder cancer, *NMIBC* Non–muscle-invasive bladder cancer, *VI-RADS* Vesical Imaging Reporting and Data System

**Table 4:** Diagnostic Performance of the VI-RADS Score for Each Reader (cut off ≥ 4)

Reader	Sensitivity (%)	Specificity (%)	Accuracy (%)	PPV (%)	NPV (%)	AUC (%)
Reader 1 (Experienced)	90.5(69.6 -98.8)	84.0(63.9—95.5)	87.0	82.6	91.3	92.3(80.5—98.1)
Reader 2 (Experienced)	95.2(76.2 -99.9)	80.0(59.3—93.2)	87.0	80.0	95.2	95.5(85.0—99.4)
Reader 3 (Experienced)	95.2(76.2 -99.9)	84.0(63.9 -95.5)	89.1	83.3	95.5	95.8(85.4—99.5)
Reader 4 (Inexperienced)	90.5(69.6 -98.8)	80.0(59.3—93.2)	84.8	79.2	90.9	91.6(79.6—97.7)
Reader 5 (Inexperienced)	90.5(69.6 -98.8)	72.0(50.6—87.9)	80.4	76.0	90.0	90.4(78.0—97.1)
Pooled experienced readers	93.7(84.5–98.2)	82.7(72.2–90.4)	87.7	82.0	93.9	94.5(89.3–97.7)
Pooled inexperienced readers	90.5(77.4–97.3)	76.0(61.8–86.9)	82.6	76.0	90.5	91.0(83.1–95.9)
Pooled all readers	92.4(85.5–96.7)	80.0(71.9–86.6)	85.7	79.5	92.6	93.2(89.2–96.1)

Note—Data in brackets show 95% confidence intervals. *AUC* Area under the ROC curve, *NPV* Negative predictive value, *PPV* Positive predictive value, *VI-RADS* Vesical Imaging Reporting and Data System

### Interobserver agreement

Interobserver agreement was excellent among the experienced readers (κ = 0.89–0.92) and inexperienced readers (κ = 0.83). Interobserver agreement between the experienced and inexperienced readers was substantial to excellent (κ = 0.79–0.88). The interobserver agreement is summarized in Table 5.

**Table 5:** Kappa Values Between Readers with Different Levels of Experience

Kappa (95% CI)/P	Reader 1 (Experienced)	Reader 2 (Experienced)	Reader 3 (Experienced)	Reader 4 (Inexperienced)
Reader 2 (Experienced)	0.92 (0.85,0.98)			
Reader 3 (Experienced)	0.89 (0.80,0.97)	0.92 (0.85,0.98)		
Reader 4 (Inexperienced)	0.88 (0.79,0.97)	0.83 (0.71,0.94)	0.79 (0.67,0.92)	
Reader 5 (Inexperienced)	0.81 (0.71,0.92)	0.84 (0.74,0.94)	0.81 (0.70,0.93)	0.83 (0.73,0.94)

*CI* Confidence intervals

## Discussion

The evaluation of muscle invasion status in bladder cancer patients after NAC is crucial for effective clinical management, given the significant impact of different tumor stage statuses. Our study demonstrated that VI-RADS exhibits robust diagnostic performance in detecting muscle invasion in bladder cancer after NAC, achieving excellent interobserver agreement across readers with diverse experience levels.

Our study demonstrated substantial to excellent agreement among all five readers (κ = 0.79–0.92), with excellent interobserver agreement observed among both experienced readers (κ = 0.89–0.92) and inexperienced readers (κ = 0.83). The substantial to excellent agreement observed across all readers further validated the previously reported high reproducibility of VI-RADS [27]. While interobserver agreement was slightly higher among experienced radiologists compared to inexperienced radiologists, the consistently high degree of inter-reader agreement among all readers supports the implementation of VI-RADS in routine clinical practice for the restaging paradigm of bladder cancer after NAC.

In our study, the pooled AUC values for VI-RADS scores in detecting MIBC were 0.945 and 0.910 for experienced and inexperienced readers, respectively. Importantly, there was no statistical difference between experienced and inexperienced readers, indicating that VI-RADS's diagnostic efficacy remains consistent regardless of the radiologists' clinical experience. This aligns with previous study results that demonstrated VI-RADS's sustained diagnostic efficacy when expanding the target population to include post-treatment patients [27]. The AUC values for the post-treatment group, primary group, and the overall group were 0.947, 0.936, and 0.945, respectively [27]. Our study establishes VI-RADS scores of ≥ 4 as the optimal cutoff for predicting muscle invasion, with pooled positive predictive and negative predictive values of 79.5% and 92.6%, respectively, for all readers. The high negative predictive value underscores VI-RADS's reliability in identifying patients without muscle invasion, reducing the risk of unnecessary invasive procedures. These findings underscore VI-RADS as a dependable and reproducible tool for detecting MIBC, irrespective of the interpreting radiologist's experience level. The consistent performance of VI-RADS in our study, coupled with its prior validation in post-treatment patients, suggests its potential applicability across a broader patient population.

Although VI-RADS scores of ≥ 4 was optimal cutoff value, tumors scored as VI-RADS 4 and 5 exhibited a high false positive rate. Our results revealed that tumors scored as VI-RADS 4 and 5 had muscle invasion risks of 55–70% and 92–100%, respectively. In contrast, prior studies evaluating muscle invasion in pre-treatment bladder cancer reported that VI-RADS 4 tumors achieved an accuracy of 90–100% in evaluating muscle infiltration [20, 24, 30], surpassing our findings. This discrepancy may be attributed to the inflammatory response in the bladder wall after NAC, complicating the assessment of muscle layer continuity.

The risk of muscle invasion ranged from 11–29% in VI-RADS 3 tumors. Although only 1–2 tumors with VI-RADS score of 3 were MIBC, the risk of muscle invasion was high, which may be caused by the small sample. Moreover, all VI-RADS 3 tumors were sessile/broad-based without a thickened inner layer, which may also be the cause of false negative results. A previous study showed that the flat appearance of the tumor is an important entity that can affect the accuracy of the VI-RADS scoring system [31]. Improving the evaluation of myometrial infiltration of bladder tumor with flat appearance is worth further exploring in the future. Additionally, it's important to highlight that two VI-RADS 3 tumors exhibited submucosal linear enhancement on DCE imaging. Of these, one tumor showed continuous submucosal enhancement, confirmed as NMIBC, while the other demonstrated discontinuous submucosal enhancement and was confirmed as MIBC by pathology. A prior study validated that submucosal linear enhancement beneath the tumor base on DCE-MRI improved the accuracy of differentiating stage T1 from stage T2 bladder urothelial carcinoma [32]. Future studies with larger sample sizes are necessary to confirm whether the continuity of submucosal linear enhancement can reduce false negatives in VI-RADS 3 scoring for bladder cancer after neoadjuvant chemotherapy (NAC). Tumors scored as VI-RADS 1 and VI-RADS 2 showed no risk of muscle invasion, therefore bladder-sparing therapy may be considered the initial choice for patients with a score ≤ 2. In clinical practice, it may be more reasonable to assess muscular infiltration using a cutoff value of ≥ 4 for patients after NAC. Radical cystectomy could be the preferred treatment option for patients with a score ≥ 4, whereas for patients with a VI-RADS score 3, treatment options should be determined by individual discussion based on their performance status and overall tumor clinical stage.

### Limitations

Several limitations are present in our study. Firstly, it was a retrospective study conducted at a single institution with a sample size, necessitating further validation through larger cohorts from prospective multicenter studies utilizing multiple scanners to confirm the stability and reproducibility of our findings. Secondly, some patients did not undergo cystectomy, potentially leading to an underestimation of bladder cancer staging based on pathological findings obtained from TURBT [33]. Thirdly, the patients in this study did not undergo TURBT for pathologic confirmation of muscle invasion before NAC. However, we retrospectively evaluated the pre-NAC multiparametric MRI of all patients and assigned VI-RADS scores. A recent meta-analysis, encompassing 20 studies with 2725 patients, reported that VI-RADS achieved commendable performance in detecting muscle invasion status in bladder cancer. The pooled sensitivity and specificity of VI-RADS were reported as 0.82 and 0.95, respectively, when applying VI-RADS ≥ 4 [28]. Given the high specificity of VI-RADS ≥ 4, Taguchi, Satoru et al. proposed a new algorithm based on VI-RADS for the management of bladder cancer [32]. According to their proposal, patients with VI-RADS ≥ 4 may omit conventional TURBT and undergo intensive treatment [32]. Therefore, the present study selected patients with VI-RADS ≥ 4 on pre-NAC MRI. Our study demonstrated that VI-RADS had robust diagnostic performance in detecting muscle invasion status in bladder cancer after NAC. This finding may eventually facilitate personalized therapeutic options for patients, rather than proceeding to cystectomy, the current standard practice after NAC.

## Conclusion

The VI-RADS demonstrated accurate assessment of muscle invasion in MIBC patients after NAC treatment, exhibiting good diagnostic performance across readers with varying levels of experience.

## Data Availability

Not applicable.

## Abbreviations

AUC: Area under receiver operating characteristic curve
CI: Confidence interval
DCE: Dynamic contrast-enhanced
MIBC: Muscle-invasive bladder cancer
MRI: Magnetic Resonance Imaging
NAC: Neoadjuvant chemotherapy
NMIBC: Non-muscle-invasive bladder cancer
TURBT: Transurethral resection of bladder tumor
T2WI: T2-weighted imaging
VI-RADS: Vesical Imaging Reporting and Data System
